# Quantification of sphingosine 1-phosphate by validated LC-MS/MS method revealing strong correlation with apolipoprotein M in plasma but not in serum due to platelet activation during blood coagulation

**DOI:** 10.1007/s00216-015-9008-4

**Published:** 2015-09-16

**Authors:** Cecilia Frej, Anders Andersson, Benny Larsson, Li Jun Guo, Eva Norström, Kaisa E. Happonen, Björn Dahlbäck

**Affiliations:** Department of Translational Medicine, Lund University, Skåne University Hospital, Inga Marie Nilssons gata 53, 205 02 Malmö, Sweden; Department of Clinical Chemistry and Pharmacology, Lund University, Skåne University Hospital, Klinikgatan 19, 221 85 Lund, Sweden; Department of Clinical Chemistry, Skåne University Hospital, Inga Marie Nilssons gata 53, 205 02 Malmö, Sweden

**Keywords:** Sphingolipid, Apolipoprotein, Mass spectrometry, Liquid chromatography

## Abstract

Sphingosine 1-phosphate (S1P) is a signalling sphingolipid affecting multiple cellular functions of vascular and immune systems. It circulates at submicromolar levels bound to HDL-associated apolipoprotein M (apoM) or to albumin. S1P in blood is mainly produced by platelets and erythrocytes, making blood sampling for S1P quantification delicate. Standardisation of sampling is thereby of great importance to obtain robust data. By optimising and characterising the extraction procedure and the LC-MS/MS analysis, we have developed and validated a highly specific and sensitive method for S1P quantification. Blood was collected from healthy individuals (*n* = 15) to evaluate the effects of differential blood sampling on S1P levels. To evaluate correlation between S1P and apoM in different types of plasma and serum, apoM was measured by ELISA. The method showed good accuracy and precision in the range of 0.011 to 0.9 μM with less than 0.07 % carryover. We found that the methanol precipitation used to extract S1P co-extracted apoM and several other HDL-proteins from plasma. The platelet-associated S1P was released during coagulation, thus increasing the S1P concentration to double in serum as compared to that in plasma. Gel filtration chromatography revealed that the platelet-released S1P was mainly bound to albumin. This explains why the strong correlation between S1P and apoM levels in plasma is lost upon the clotting process and hence not observed in serum. We have developed, characterised and validated an efficient, highly sensitive and specific method for the quantification of S1P in biological material.

**Electronic supplementary material:**

The online version of this article (doi:10.1007/s00216-015-9008-4) contains supplementary material, which is available to authorized users.

## Introduction

Sphingosine 1-phosphate (S1P) is a sphingolipid with pleiotropic functions [1, 2]. Extracellular actions of S1P are mediated via binding and stimulation of five different G-coupled receptors, S1P_1–5_ [3]. In circulation, S1P is carried by apolipoprotein M (apoM) in the lipoproteins and by albumin; approximately 60 % is normally present in high-density lipoproteins (HDL), 10 % in low-density lipoproteins (LDL) and 30 % is bound to albumin [4–6]. S1P is present in plasma in the submicromolar range and can be produced by platelets, erythrocytes and endothelial cells. Platelets store S1P in their plasma membrane and α-granules and S1P release requires platelet activation [7]. However, platelets are not the main source of S1P in plasma and thrombocytopenic mice have normal S1P levels [8]. Erythrocytes, which are the most important source of plasma S1P, can phosphorylate sphingosine to generate as well as store S1P but cannot produce S1P de novo [9, 10]. These features make the handling of blood samples taken for S1P analysis important and delicate; however, guidelines for and knowledge on pre-analytical standardisation are limited [11, 12].

Different methods have been used to extract S1P from plasma, e.g. single-phase extraction [13], solid-phase extraction using small gel filtration columns [14], one-step extraction [15–17], two-step extraction [16, 18–22] and methanol precipitation [23–26]. Earlier methods using thin layer chromatography (TLC) showed poor recovery efficiencies and complex sample preparation using radioactive substances [19, 21]. Later on, high-performance liquid chromatography (HPLC) was introduced to improve automation, separation and resolution. However, S1P is difficult to separate in chromatographic systems because of its zwitterionic properties. Derivatisation with fluorescent molecules like naphthalene-2,3-dicarboxaldehyde (NDA) [16] or *o*-phthalaldehyde (OPA) [27] followed by HPLC and detection by fluorescent emission overcome the difficulties with the zwitterionic properties. However, the derivatisation techniques are time consuming and can cause unspecific binding of the fluorescent dye to other targets thereby disturbing the detection of S1P. HPLC coupled to tandem mass spectrometry (LC-MS/MS) provides high resolution and high selectivity and is the most commonly used method for quantifying S1P [23–25, 20, 18, 26, 22]. Due to the complex chemistry of S1P, carryover between sample injections has been identified as a problem. Berdyshev, E.V. et al. reported 10.5 % carryover, which they solved with bisacetylation of the amino group [15]. Other approaches to avoid carryover have been washing the injection needle several times between injections or washing with methanol between each sample analysis [22, 23]. However, most published studies do not address this issue. By optimising the LC system, we have reduced carryover to 0.07 %.

We describe a highly sensitive and specific validated method for sample handling and quantification of S1P in plasma and serum using deuterium-marked S1P as internal standard (IS), a quick methanol precipitation and a selective LC-MS/MS [28] analysis with less than 0.07 % carryover. We demonstrate that apoM correlates with S1P only in platelet-poor plasma (PPP) but not in platelet-rich plasma (PRP) or serum and that S1P released from platelets during blood coagulation mainly binds to albumin and not to apoM.

## Materials and method

### Materials

S1P (d-*erythro*-sphingosine-1-phosphate), d7S1P (d-*erythro*-sphingosine-d7-1-phosphate) and C17S1P (d-*erythro*-sphingosine-1-phosphate C17 base) were from Avanti Polar Lipids (Alabaster, USA). Methanol hypergrade was from Merck (Darmstadt, Germany). Formic acid and essentially fatty acid and globulin-free bovine serum albumin (BSA) were from Sigma-Aldrich (St. Louis, USA). The reversed-phase C18 column (XSelect CSH XP C18 130 Å, 2.5 μm, 2.1 mm × 50 mm) was from Waters (MA, USA). The 96-well polystyrene plates used for sample injection were from Porvair Sciences (Leatherhead, UK). Tubes for blood collection were from BD, Plymouth, UK (citrate: blue REF 367714, lithium-heparin: green REF 368497, EDTA: purple REF 368499, serum: red REF 367614). The LC-MS/MS system consisted of a Shimadzu Prominence HPLC system CBM20Alite controller with two Shimadzu LC20ADXR pumps, SIL-20AC autosampler, CTO-20AC column-oven and a Rack-changer–C coupled to a triple quadropole mass spectrometer API 4000 from Sciex (Framingham, USA). Samples were ionised using electrospray ionisation (ESI). Results were calculated by Analyst^®^ software version 1.6 from AB Sciex (Framingham, USA).

### Centrifugation and blood sampling

Citrate-plasma, lithium-heparin (Li-hep.)-plasma, EDTA-plasma and serum were collected from 15 healthy individuals (permission by the local ethical committee at Lund University). Citrate-plasma was centrifuged directly after collection at 300*g* for 15 min to generate platelet-rich plasma (PRP) and at 1000*g* for 10 min, 2000*g* for 10 min, 2000*g* for 20 min and 20,000*g* for 20 min to obtain platelet-poor plasma (PPP). Serum was left at room temperature for 1 h to allow the blood to clot. Li-hep-plasma, EDTA-plasma and serum were centrifuged at 2000*g* for 20 min and 20,000 *g* for 20 min whereafter the samples were stored at −80 °C until analysis. Quantification of platelets is described in the Electronic Supplementary Material (ESM). ApoM was quantified as previously described [29].

### Stock and working solutions

S1P was dissolved in methanol to obtain a stock solution of 1 mM. Calibration samples (CS) were prepared by diluting the stock in 4 % BSA dissolved in water to obtain concentrations of 0.0037, 0.011, 0.033, 0.1, 0.3 and 0.9 μM. Aliquots were stored at −80 °C and thawed directly before analysis. Samples containing 0.033, 0.1 and 0.9 μM S1P were used as quality controls (QC) in every run. The C17S1P and d7S1P were dissolved in methanol to obtain stock solutions of 200 nM and stored at −20 °C.

### S1P extraction and sample preparation

Plasma and serum samples (10 μL) were diluted with 55 μL TBS (50 mM Tris-HCl pH 7.5, 0.15 M NaCl). Precipitation solution (200 μL methanol containing 20 nM IS) was added to 65 μL CS, QC or the TBS-diluted plasma and vortexed at maximum speed for 30 s. Samples were centrifuged at 17,000*g* for 2 min after which 150 μL of supernatants was transferred to a 96 well polystyrene plate, and 5 μL was injected for analysis by LC-MS/MS. The protein content of the extract was analysed by running plasma and methanol-extracted plasma on SDS-PAGE gel followed by silver staining and western blotting using an in-house anti-human apoM antibody (rabbit polyclonal anti-human apoM nr 0101), raised against recombinant human apoM (residues 22–188) and characterised as previously described [30, 31], anti-human apoAII (Nordic Biosite, Täby, Sweden), and anti-human apoA1, anti-human apoB100 and anti-human apoE (all from Dako, Glostrup, Denmark).

### LC-MS/MS analysis

Analytes were separated on the reversed-phase C18 column using a gradient of buffers A (water/methanol/formic acid 97/2/1 (*v*/*v*/*v*)) and B (methanol/acetone/water/formic acid 68/29/2/1 (*v*/*v*/*v*/*v*)) with a flow rate of 0.4 mL/min. The column was maintained at 60 °C and total time of analysis was 10 min. Ionisation of analytes was made by ESI operating in positive ionisation mode, and the scanning mode was multiple reaction monitoring (MRM). To determine the optimal ion source and MRM parameter settings, S1P and IS were injected post-column into the mass spectrometer and full-scan product-ion spectra were obtained (MRM settings are described in Table 1). To monitor the level of contamination of the LC column and mass spectrometer with blood lipids, the three carbon C^13^ isotopes of palmitoyl-oleoyl-phosphatidylcholine were analysed on *m*/*z* transition 763/185. Results were integrated and calculated using linear regression by the Analyst^®^ software. The 10 % dilution factor from the citrate solution in the citrate tubes was compensated for in the calculation of S1P concentration.

**Table 1:** MRM settings. The specific MRM settings for each ion are presented in the table. The specific tuning parameters for the MS/MS (Sciex API 4000) were ion source temperature 550 °C, ion source voltage +5500 V, nebulizer gas setting 50, drying gas setting 60, curtain gas setting 20, entrance potential +10 V and nitrogen collision chamber gas pressure 6 psi

Q1	Q3	Dwell (ms)	DP	CE	CXP
380.3	264.5	200	81	27	16
380.3	82.1	200	81	45	14
387.3	271.1	200	91	27	14
387.3	82.1	200	91	47	8
763.6	185.3	30	110	43	12

### Method validation

#### Ion suppression test

S1P was continuously injected post-column to the mass spectrometer to retain a stable S1P signal, and then an ethanol-extracted plasma sample containing the C17S1P was injected pre-column. A countersink of the stabilised S1P signal caused by components in the plasma sample was an indication of ion suppression at that retention time. The ion suppression analysis was made on five individual samples, one of which was serum.

#### Carryover

After analysis of the highest calibration sample (0.9 μM), a blank was injected (mobile phase A or TBS buffer) and the S1P peak measured. The degree of carryover was estimated by dividing the S1P peak in the blank with that of the calibration sample from seven individual experiments.

#### IS purity

IS purity was analysed by analysing TBS extracted with methanol containing d7S1P.

#### Linearity

Linearity was analysed by plotting the peak area of the analyte divided with the peak area of IS (*y*) versus the spiked concentration of S1P (*x*). The correlation coefficient (R^2^) was calculated as the value of the joint variation between *x* and *y*.

#### Recovery

Recovery was evaluated by spiking plasma samples with known amounts of S1P and calculated as [(final concentration − initial concentration)/added concentration * 100].

#### Accuracy and precision

To evaluate precision and accuracy, QC samples were analysed within runs (intra-run validation) at 10 replicate analyses and between runs (inter-run validation) at 10 different occasions. Accuracy was calculated as the mean of measured concentration/spiked concentration * 100, and precision was calculated using the coefficient of variation CV % as standard deviation/mean of measured concentration * 100.

#### Stability and selectivity

The methodology for stability and selectivity experiments is described in the ESM.

### Gel filtration of serum and plasma

Citrate-plasma or serum (500 μL) pooled from 10 healthy individuals was applied to a Superose 6 10/300 GL column connected to a ÄKTA AVANT (GE Healthcare, Uppsala, Sweden). Gel filtration chromatography was performed in TBS at a flow rate of 0.4 mL/min, and 30 fractions each containing 300 μL were collected. ApoM, S1P and albumin were measured in the fractions using methods described above and with the QuantiChrom BCG Albumin Assay Kit (Bioassay Systems, USA).

### Statistical analysis

Statistical significance of the differences between groups was measured by Friedman’s test with Dun’s multiple comparison. Correlation analysis was made according to Spearman. S1P content in gel filtration peaks was measured by analysis of area under the curve. Statistics were calculated using GraphPad Prism 4.0 (GraphPad Software, La Jolla, CA, USA).

## Results

### Optimising peak shape and LC column

**Fig. 1:**
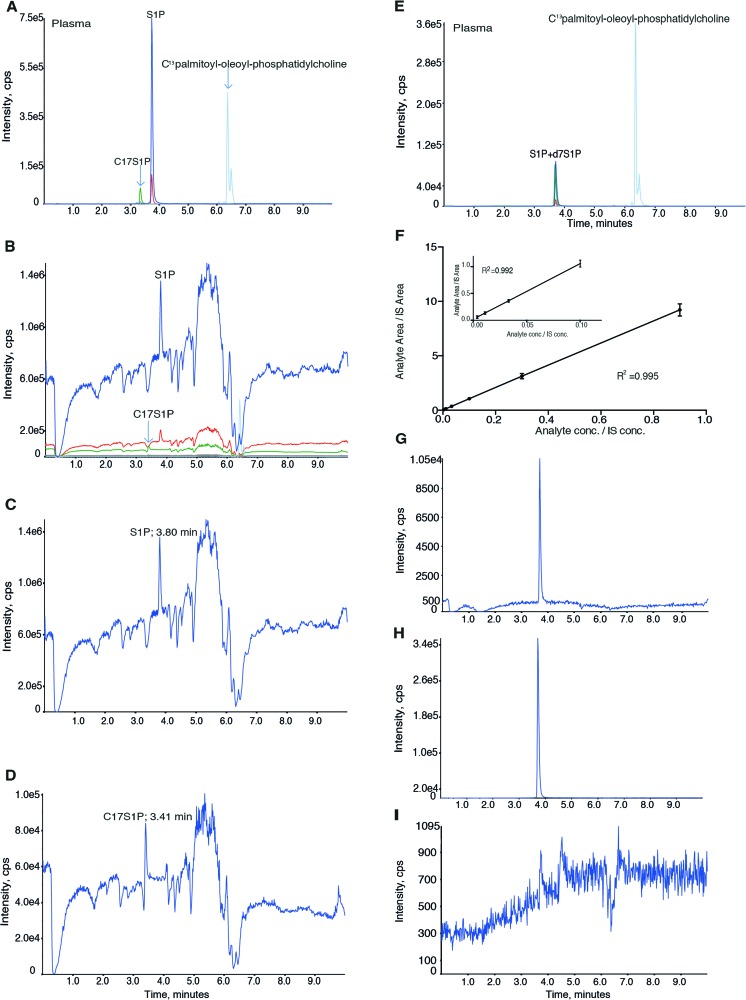
Evaluation of ion suppression and elution profile for C17S1P, S1P and deuterium-marked S1P. **A** Elution profile of S1P extracted from human plasma with C17S1P as IS. **A**, **B** The *dark blue* and *red traces* represent the quantification (*m*/*z* 380/264) and qualification ions for S1P (eluted after 3.8 min), respectively. The *green* and *grey traces* represent the quantification (*m*/*z* 366/250) and the qualification ions (*m*/*z* 366/82) for C17S1P (eluted after 3.4 min), respectively. The *light blue line* represents carbon C^13^ isotopes of palmitoyl-oleoyl-phosphatidylcholine (*m*/*z* 763/185) that elute after 6–7 min. **B**–**D** Ion suppression analysis in human plasma. **B** Elution timepoints for C17S1P, S1P and palmitoyl-oleoyl-phosphatidylcholine, **C** quantification ion of S1P (*m*/*z* 380/264) and **D** quantification ion for C17S1P (*m*/*z* 366/250). **E** Elution profile of S1P extracted from human plasma with d7S1P as IS. The chromatogram shows ions for S1P and d7S1P both eluting after 3.7 min and carbon C^13^ isotopes of palmitoyl-oleoyl-phosphatidylcholine, eluting after 6–7 min. **F** Plotted standard curve of CS. **G** S1P signal in 0.011 μM CS (LLOQ); **H**, **I** carryover evaluation. Representative chromatograms of 0.9 μM CS (**H**) and blank (**I**)

S1P contains a hydrophobic C18 aliphatic chain and hydrophilic inorganic phosphoric acid group giving S1P amphipathic properties. In addition, S1P has a primary amino group and a hydroxyl group bound to the aliphatic chain giving S1P zwitterionic properties. These properties are important to consider when optimising chromatographic separation of S1P since it will affect the intermolecular forces in the LC column. We tested a variety of different LC columns, HILIC and different reversed-phase columns such as C8 and C18, but all gave rise to distinct peak tailing, which is an indication of carryover (data not shown). A C18 column from Waters using the Charged Surface Hybrid Technology generated a positively charged surface at low pH. This column together with a buffer system consisting of water-based mobile buffer A and methanol/acetone-based buffer B, both with a pH between 1 and 2, gave a better peak shape without peak tailing and adequate elution time of 3.7 min (Fig. 1E, ESM Fig. S1A and S1B).

### C17S1P versus deuterium-marked S1P as IS

Initially, we used C17S1P as IS since it is the most commonly used IS for S1P quantification. When analysing methanol extract from human plasma and serum, C17S1P eluted after around 3.4 min and S1P after 3.8 min (Fig. 1A). Since the C17S1P and S1P did not co-elute from the LC column (Fig. 1A), we evaluated for ion suppression at their respective elution timepoints in four individual plasma samples and one serum sample. We observed a similar ion suppression in all analysed samples at the elution time of C17S1P but not at that of S1P (representative chromatogram Fig. 1B–D). Therefore, C17S1P is not an optimal IS as the matrix effect may affect quantification and thereby increase variation. We instead tried deuterium-marked S1P (d7S1P) as IS, which has the advantage that it co-elutes with S1P from the LC system (Fig. 1E, ESM Fig. S1A and S1B). To evaluate IS purity, TBS was extracted with methanol containing IS. No peak at the retention time of S1P larger than noise could be detected (ESM Fig. S1C and S1D).

### Validation results

#### Linearity and LLOQ

The calibration curve was linear, *R*^2^ = 0.995 (*n* = 10), showing excellent correlation between signal (*y*) and concentration (*x*) (Fig. 1F). The second lowest CS, 11 nM (Fig. 1G), fulfilled the requirements to be the LLOQ (signal to noise ratio more than 5 and precision and accuracy within 20 and 80–120 %, respectively) (ESM Table S2).

#### Carryover

We analysed the carryover effect of S1P by injecting blank (Fig. 1I) after the injection of 0.9 μM CS (Fig. 1H), and the S1P signal in the blank was divided with the signal in the 0.9 μM sample. The mean carryover effect from seven individual experiments was 0.065 ± 0.05 %, which can be considered negligible.

#### Specific HDL-apolipoproteins present in the methanol extract

**Fig. 2:**
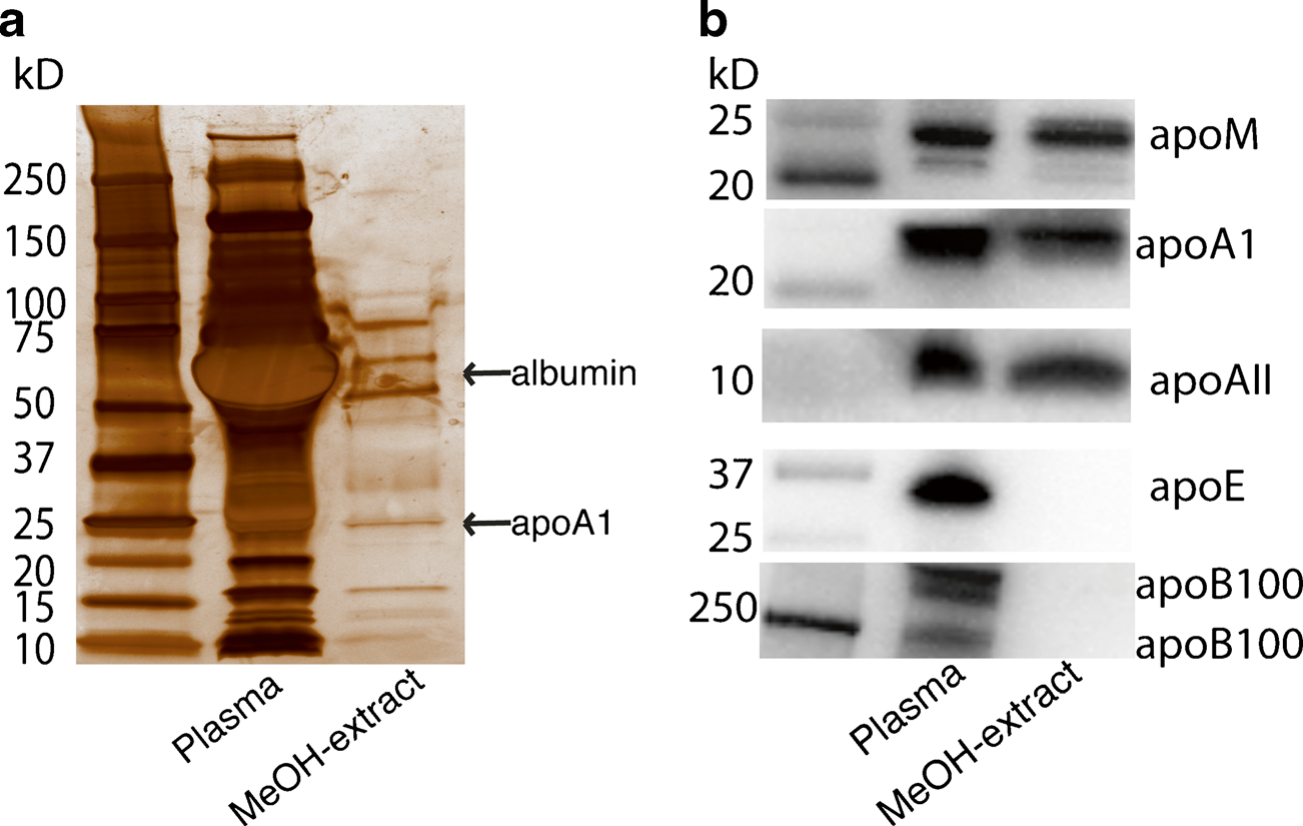
HDL-proteins in methanol extract. **A**, **B** Citrate-plasma was extracted by methanol precipitation and compared to unextracted citrate-plasma. **A** Total protein analysis by silver staining. **B** Apolipoprotein content analysed by western blotting

When spiking pure methanol with S1P in the submicromolar range, we obtained S1P levels lower than the predicted ones. However, when S1P was added to an albumin-containing solution, the measured values were the same as those predicted (data not shown). Since we use methanol precipitation when extracting S1P from samples, we wanted to investigate if any of the carrier proteins, e.g. apoM or albumin, were co-extracted with S1P during the methanol precipitation and thereby stabilising S1P in the extract. After methanol extraction, both albumin and HDL-proteins were present in the extract as visualised by silver staining and western blotting (Fig. 2A, B). When analysing the presence of apolipoproteins in the extract, we found that the HDL-specific apolipoproteins apoM, apoA1 and apoAII were present in the extract, whereas apoB100 and apoE were not (Fig. 2B).

#### Selectivity

When plasma or 0.9 μM QC was treated with charcoal, S1P levels decreased by 82.9 and 100 %, respectively (ESM Table S1). To further analyse selectivity, HDL and HDL depleted of the S1P carrier apoM (see ESM: Methods) were analysed. The apoM levels dropped by 100 % when apoM was depleted from HDL (ESM Fig. S2A and S2B). The signal for S1P decreased by 93.4 % in the apoM-depleted HDL compared to total HDL (ESM Fig S2A, right panel). Finally, in the selectivity validation, 4 % BSA, TBS and mobile buffer A were analysed. The S1P concentration was very low (0.5 nM) in the 4 % BSA and below detection level in TBS and mobile buffer A (ESM Table S1).

#### Recovery

Recovery was calculated by spiking six plasma samples with known amounts of S1P. Recovery efficiencies varied between 95.2 and 111 % (Table 2). Mean recovery was 103 %

**Table 2:** Recovery of S1P in spiked plasma samples. Known amounts of S1P were added to citrate-plasma (*n* = 6). Experiments were carried out in duplicate with one analysis for each experiment. Recovery was calculated as follows [(final concentration − initial concentration)/added concentration * 100]

Sample no.	Unspiked (μM)	Spiked (μM, mean ± SD)	Measured difference (μM)	Expected difference (μM)	Recovery (%)
1	0.628	1.26 ± 0.009	0.633	0.570	111
2	0.838	1.41 ± 0.028	0.572	0.570	100
3	0.748	1.29 ± 0.060	0.542	0.570	95.2
4	0.510	1.11 ± 0.046	0.602	0.570	106
5	0.696	1.28 ± 0.018	0.585	0.570	103
6	0.838	1.42 ± 0.009	0.585	0.570	103

#### Stability of S1P at room temperature and after repeated freeze and thaw cycles

S1P was stable when leaving citrate-plasma, serum and QC samples at room temperature for 0–24 h (ESM Fig S3A–S3C), in agreement with results previously reported for EDTA-plasma [17]. When subjecting S1P to repeated freeze and thaw cycles, S1P decreased by 6 % in citrate-plasma after being frozen once but was unaffected by further freeze-thaw cycles (ESM Fig S3D and S3E).

#### Accuracy and precision

Values for accuracy and precision are summarised in ESM Table S2. Accuracy and precision for intra- and inter-validation were below 12 %, which is within recommended limits.

#### S1P and apoM in platelet-rich and platelet-poor plasma

**Fig. 3:**
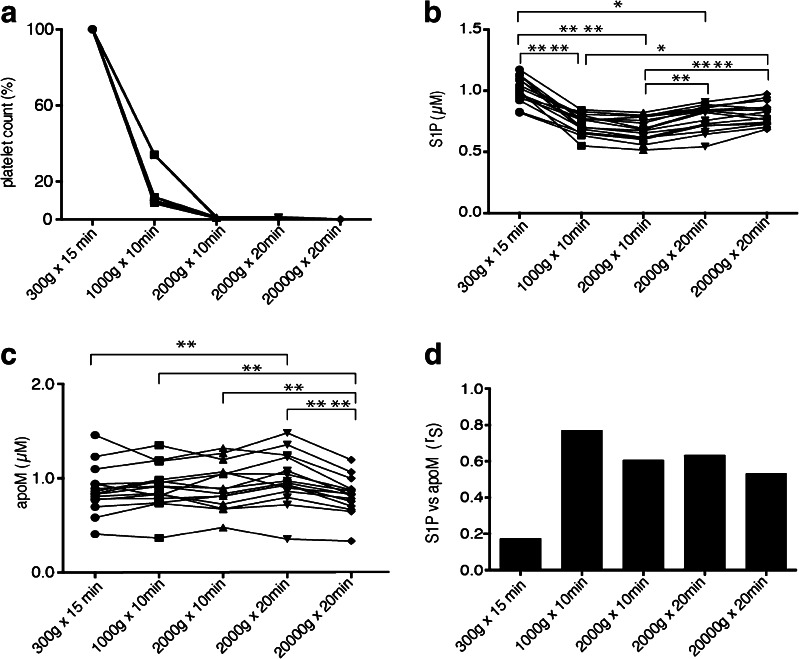
Platelet contamination affecting S1P analysis and the S1P correlation to apoM. Citrate-plasma was collected from 15 healthy individuals and centrifuged at different protocols. **A** Platelet concentration was measured by flow cytometry, **B** S1P by LC-MS/MS and **C** apoM by ELISA. **D** Correlation analysis between S1P and apoM. Results are plotted as individual values. Statistical analysis between groups was made by Friedman’s test, and correlation was calculated by Spearman’s rank correlation coefficient (*r*_S_). *p < 0.05; **p < 0.01, ****p < 0.0001

To investigate whether different centrifugation protocols leave varying amounts of platelets in the plasma and thus an erroneous high S1P concentration, citrate-plasma was collected from 15 healthy individuals and subjected to different centrifugation protocols. Platelets were measured in the first five individuals to determine how many platelets were left after each centrifugation step (Fig. 3A). S1P was significantly higher in PRP (300*g* for 15 min) compared to PPP (1000*g* for 10 min, 2000*g* for 10 min and 2000*g* for 20 min) (Fig. 3B). However, there was a noteworthy significant increase of the S1P concentration when the plasma was centrifuged at 2000*g* for 20 min and more (Fig. 3B). ApoM demonstrated a slight but significant increase in plasma after centrifugation at 2000 *g* for 20 min and a slight decrease after 20,000 *g* for 20 min as compared to plasma that was centrifuged at lower speed and time (Fig. 3C). As the S1P concentration was much higher in PRP compared to PPP and the apoM levels were relatively stable, the correlation between apoM and S1P in PRP was much lower than that observed in PPP (Fig. 3D). Even though there was a slight variation of both apoM and S1P levels between the different centrifugation protocols in PPP (from 1000*g* for 10 min to 20,000*g* for 20 min), the correlation between apoM and S1P between these groups was similar (Fig. 3D).

#### S1P and apoM in different types of plasma and serum

**Fig. 4:**
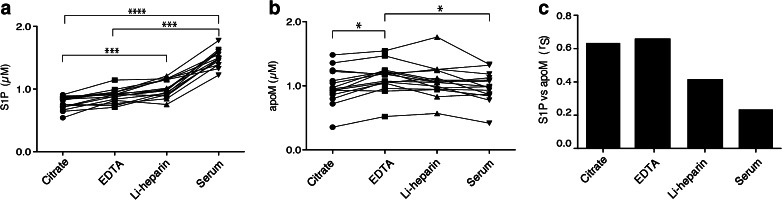
S1P and apoM concentrations in different plasma types and in serum. Citrate-, Li-hep- and EDTA-plasma and serum were collected from 15 healthy individuals and centrifuged for 2000*g* in 20 min. **A** S1P was analysed by LC-MS/MS. **B** apoM was analysed by ELISA; **C** correlation analysis between S1P and apoM. Results are plotted as individual values. Statistical analysis between groups was made by Friedman’s test, and correlation was calculated by Spearman’s rank correlation coefficient (*r*_S_). *p < 0.05; ***p < 0.001, ****p < 0.0001

The effect of different anticoagulants and serum on S1P levels has been evaluated before [12], but the correlation between apoM and S1P has not been assessed. S1P levels in citrate-, Li-hep-, and EDTA-plasma and serum were analysed by collecting samples from 15 healthy individuals. The plasma S1P concentration ranged between 0.5 and 1.2 μM, in agreement with previously reported results [32, 23, 17], whereas the serum S1P levels were between 1.4 and 1.8 μM, which is higher than those reported before (Fig. 4A) [12]. S1P levels in Li-hep- and EDTA-plasma were slightly higher than that in citrate-plasma (Li-hep *p* < 0.05). Serum S1P levels were significantly higher than S1P levels in citrate- and EDTA-plasma but not significantly different from that in Li-hep-plasma (Fig. 4A). These results were consistent after centrifuging the samples additionally at 20,000*g* for 20 min (data not shown). ApoM was measured in the same samples and found to be slightly lower in serum and citrate-plasma as compared to EDTA-plasma (*p* < 0.05) (Fig. 4B). ApoM and S1P levels correlated most strongly in EDTA-plasma (*r* = 0.66, *p* = 0.0089) and citrate-plasma (*r* = 0.63, *p* = 0.013), whereas there was no significant correlation in Li-hep (*r* = 0.42, *p* = 0.12) and serum (*r* = 0.23, *p* = 0.4) (Fig. 4C).

#### S1P released during blood clotting binds mainly to albumin and not to apoM

**Fig. 5:**
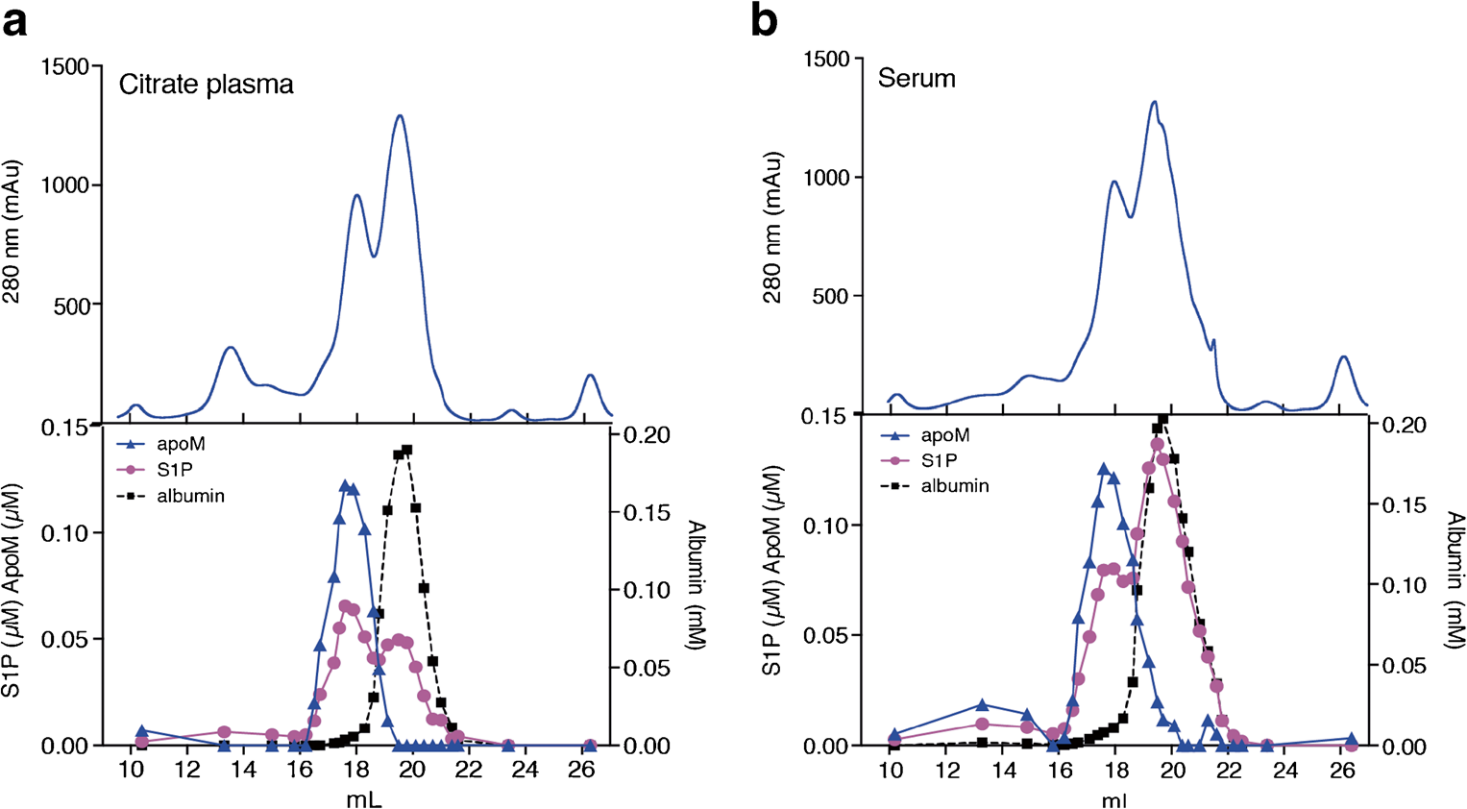
S1P released during blood coagulation binds to albumin. Citrate-plasma or serum pooled from 10 individuals (total volume 500 μL) was subjected to gel filtration chromatography. S1P was extracted from the collected fractions and analysed in LC-MS/MS. ApoM was measured by ELISA and albumin was measured by a commercial kit. Area under the curve was calculated to evaluate the S1P content eluted with either apoM or albumin

Since the S1P concentration was so much higher in serum than in citrate-plasma, we were interested in studying whether the S1P that is released during coagulation is taken up by apoM or if it binds to albumin. Plasma and serum were applied to gel filtration chromatography and fractions analysed for S1P, apoM and albumin. In citrate-plasma, approximately 60 % of total S1P co-eluted with apoM and 40 % with albumin (Fig. 5A). However, in serum, opposite results were obtained, with 35 % of total S1P co-eluting with apoM and 65 % with albumin (Fig. 5B).

## Discussion

We present a highly selective LC-MS/MS method for the quantification of S1P, using deuterium-marked S1P as internal standard. The method, which includes a simple sample preparation, has negligible carryover and enhanced performance as compared to previously published methods.

We observed that S1P spiked into pure methanol was unstable, whereas S1P was stable both when spiked in albumin-containing solution and in methanol extracts of serum/plasma. This was likely explained by the presence of apoM and other HDL-proteins in the methanol extract that could serve to stabilise S1P. Presumably, the proteins lose their tertiary structures in the methanol phase, thus increasing the accessibility of S1P in the LC-MS/MS analysis step.

Optimally, the matrix used for S1P calibration would be plasma, where S1P is present normally. However, efforts to remove S1P from plasma with activated charcoal did not result in complete depletion of S1P. Instead, an albumin-based matrix was used, which gave excellent linearity and low background. Recovery analysis yielded 95–111 % recovery of spiked S1P, demonstrating that S1P in plasma could be accurately quantified. Carryover effects of S1P between injections is a known phenomenon [15, 23, 22]. We were able to decrease the carryover to less than 0.07 %. This is most likely due to repelling forces between the positively charged surface of the LC column and the positively charged amino group on S1P, which made S1P bind less strongly to the stationary phase.

Both platelets and erythrocytes store and release S1P [33, 7, 9, 10, 32]. In a contracted blood clot, erythrocytes adopt a polyhedral shape in a densely packed tessellated configuration with platelets and fibrin lining the exterior [34]. Similar polyhedral erythrocyte structures also arise when anticoagulated blood is centrifuged at ≥1000*g* [34]. This extensive cellular re-arrangement may affect S1P release from the erythrocytes and possibly explains the higher S1P we noticed in plasma samples that had been subjected to the high-speed centrifugations. However, sufficient centrifugal forces are needed to ensure proper removal of platelets, as platelet contamination increases the measured S1P. Centrifugation of citrate-plasma at 1000*g* for at least 10 min was required to remove ≥90 % of the platelets. Since S1P slightly increased and apoM slightly decreased upon increasing the centrifugation speed and time, the best correlation between the two parameters was seen after centrifugation of plasma at 1000*g* for 10 min or 2000*g* for 10 min, which are suitable standard protocols for analysis of apoM and S1P plasma samples. In addition, the correlation between S1P and apoM was strongest in EDTA- and citrate-plasma, which should therefore preferentially be chosen for apoM and S1P analysis in biological samples.

By comparing S1P elution profiles on gel filtration chromatography of citrate-plasma and serum, we observed that most S1P released during blood clotting was preferentially bound to albumin rather than to apoM in HDL. This is consistent with a report showing that platelets release more S1P in the presence of albumin than in the presence of lipoproteins [4]. However, whether this is due to saturation of apoM or the requirement of a specific uptake mechanism for S1P entry into apoM is unknown. Since S1P bound to HDL has been suggested to have different functions than S1P bound to albumin [35], it is possible that S1P released during blood clotting has a distinct biological function from S1P circulating in HDL. S1P have several functions in coagulation [1, 36]. However, the role of the different plasma pools of S1P, i.e. the S1P carried by apoM in HDL and that carried by albumin, in the homeostasis of blood coagulation is yet to be clarified.

In conclusion, we have developed a highly sensitive and specific LC-MS/MS method for measuring S1P in biological samples, providing in addition a standardised sample collection and preparation protocol. As many parameters may affect S1P release into the samples after the actual blood collection, standardised sample handling procedures need to be used to obtain reproducible results when comparing both inter-study variation as well as variations between samples collected from different patient groups.

## Electronic supplementary material

ESM 1 (PDF 623 kb)
