# Supplementary material for: Quantification of sphingosine 1-phosphate by validated LC-MS/MS method revealing strong correlation with apolipoprotein M in plasma but not in serum due to platelet activation during blood coagulation
Source: Anal Bioanal Chem. 2015 Sep 16;407:8533–42. doi: 10.1007/s00216-015-9008-4 (PMC4635185; doi:10.1007/s00216-015-9008-4)
Supplement: Supplementary file 1 — (PDF 623 kb) [file 216_2015_9008_MOESM1_ESM.pdf]

**Analytical and Bioanalytical Chemistry**

**Electronic Supplementary Material**

# Quantification of sphingosine 1-phosphate by validated LC-MS/MS method revealing strong correlation with apolipoprotein M in plasma but not in serum due to platelet activation during blood coagulation

Cecilia Frej, Anders Andersson, Benny Larsson, Li Jun Guo, Eva Norström,
Kaisa E. Happonen, Björn Dahlbäck

## Materials and methods

### *Stability*

Citrate plasma and serum were collected from 3 individuals, centrifuged 2000*g* for 20 min and one aliquot was immediately frozen at -80 °C. The remaining volume was then transferred to fresh tubes and centrifuged 2000*g* for 20 min to completely clear the supernatant from platelets and 50 μL from each sample were frozen at -80°C. The remaining plasma was divided in two aliquots per experimental timepoint and stored at RT for 0.5, 1, 3 and 24 hours followed by freezing at -80°C. The same stability experiments were made on the QC samples. To evaluate freeze and thaw stability, QC and citrate plasma were thawed unassisted and frozen at -80°C repeatedly for 4 cycles and compared to samples not frozen or sampled frozen once.

### *Selectivity.*

To evaluate the selectivity, S1P was removed from plasma or 0.9 uM QC by addition of 100 mg activated charcoal to 1000 μL sample and incubated for 16 hours at RT, followed by 2 x centrifugation at 10000*g* for 10 min and filtration through a membrane with pore size 0.22 μm. To further analyse selectivity, HDL, apoM-depleted HDL, 4 % BSA, TBS and mobile buffer A were analysed.

### *Preparation of apoM-depleted HDL*

Human plasma (300 mL) pooled from 5 healthy blood donors (obtained from blood bank at the hospital in Växjö, Sweden) was mixed with 300 mL saturated NaBr-solution. The plasma-mix was centrifuged in centrifugation tubes (#342414, Beckman Coulter, Brea, CA, USA) at 45 000 rpm for 24 hours at 12 °C in a Beckman Coulter rotor 70.i. The lipoprotein fraction was collected at the top of the tube by aspiration

with a needle. The lipoproteins were separated by gel-filtration (Sepharose CL-6B column 2.5 x 70 cm coupled to an ÄKTA Avant, GE Healthcare, Uppsala, Sweden) with flow rate 0.5 ml/min collecting 2 mL per fraction. The HDL-fraction was depleted of apoM using tandem connected affinity chromatography columns coupled with two in-house monoclonal anti-human apoM antibodies (M23 and M58 coupled to HiTrap columns). ApoM and apoA1 levels were analysed by ELISA (performed as previously described (1, 2)) and western blotting using a polyclonal rabbit anti-human apoM (made in house) and polyclonal rabbit antihuman apoA1 (Dako, Denmark).

*Platelet count*

Platelets were analysed using flow cytometry (FC500, Beckman Coulter, Brea, CA, USA). Thresholds for forward scatter and side scatter were set to 2. Forward scatter, side scatter and fluorescence channels were set at logarithmic gain. 5 µl PRP or 20 µl of supernatant from the following centrifugation steps; 1000*g* for 10 min, 2000*g* for 10 min, 2000*g* for 20 min and 20000*g* for 20 min were stained with 5 µl CD61-PE for 30 min and CD61-positive platelets were collected and compared to a known concentration of calibration beads (Flow count, Beckman Coulter). The flow cytometry data were analysed using the software Kaluza (Beckman Coulter).

## References


1. Axler O, Ahnström J, Dahlbäck B. An ELISA for apolipoprotein M reveals a strong correlation to total cholesterol in human plasma. J Lipid Res 2007;48(8):1772-1780.
2. Kumaraswamy SB, Linder A, Akesson P, et al. Decreased plasma concentrations of apolipoprotein M in sepsis and systemic inflammatory response syndromes. Crit Care 2012;16(2):R60.

**Table S1** Selectivity for S1P in LC-MS/MS method. Human plasma (n=3) and 0.9 μM QC were treated with charcoal to strip out the S1P. Experiments for citrate plasma were carried out in duplicates and experiments for QC in triplicates with one analysis for each experiment. In addition, 4 % BSA, TBS, mobile buffer A were analysed with two analysis per sample

| Sample | Before charcoal treatment. (uM mean ± SD) | After charcoal treatment. (uM mean ± SD) | Decrease in % |
|---|---|---|---|
| Citrate plasma | 0.32 ± 0.06 | 0.055 ± 0.03 | 82.9 |
| 0.9 uM QC | 0.951 ± 0.014 | 0.00 ± 0.000 | 100.0 |
| 4 % BSA | 0.0005 ± 0.001 | | |
| TBS buffer | 0 ± 0.000 | | |
| Mobile buffer A | 0 ± 0.000 | | |

**Table S2** Precision and accuracy for the S1P quantification method. Accuracy and precision were calculated for intra run validation after 10 replicates of QC sample within the same experimental run and for inter run validation for QC samples after 10 individual experimental runs. LLOQ was defined after 10 individual replicate analysis to be the second lowest calibration standard (11nM). All values were within 15 %

| Expected (μM ) | Obtained (μM, mean) | SD | Accuracy (%) | Precision (CV %) |
|---|---|---|---|---|
| Intra run validation | | | | |
| 0.033 | 0.0336 | 0.000425 | 102 | 1.26 |
| 0.100 | 0.106 | 0.00220 | 106 | 2.07 |
| 0.900 | 1.00 | 0.0147 | 111 | 1.46 |
| Inter run validation | | | | |
| 0.033 | 0.0323 | 0.00217 | 97.8 | 6.73 |
| 0.100 | 0.103 | 0.00533 | 103 | 5.19 |
| 0.900 | 0.972 | 0.0548 | 108 | 5.65 |
| 0.011 LLOQ | 0.0101 | 0.00151 | 91.6 | 14.9 |

Supplemental figure 1

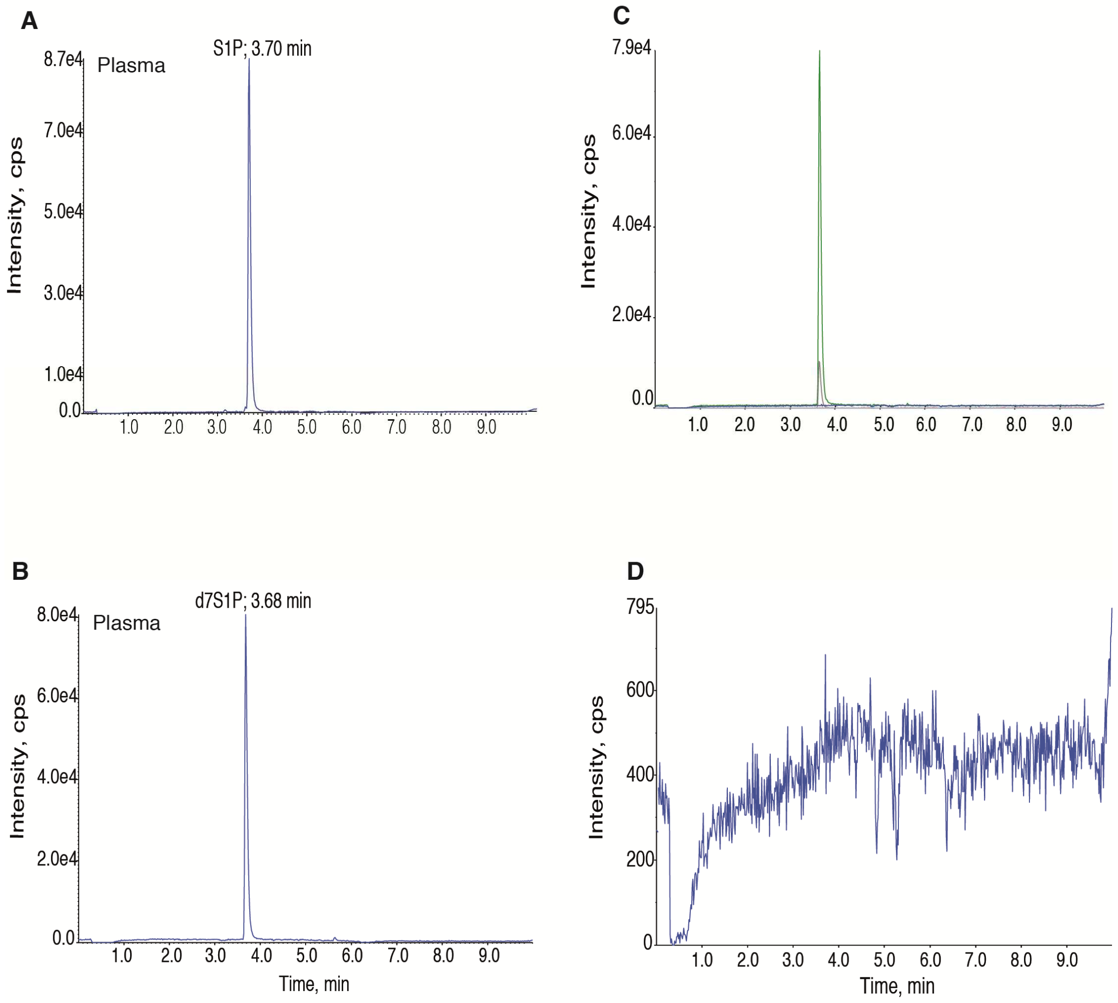


**Fig. S1 Elution profile for S1P and d7S1P.** Elution profile of S1P extracted from un-spiked human plasma with d7S1P as IS. A), S1P (*m/z* 380/264) eluted at 3.70 minutes B), d7S1P (*m/z* 387/271) eluted together with S1P at 3.68 minutes. C-D), TBS buffer precipitated in the presence of d7S1P C) signal for d7S1P and D) signal for S1P

Supplemental figure 2

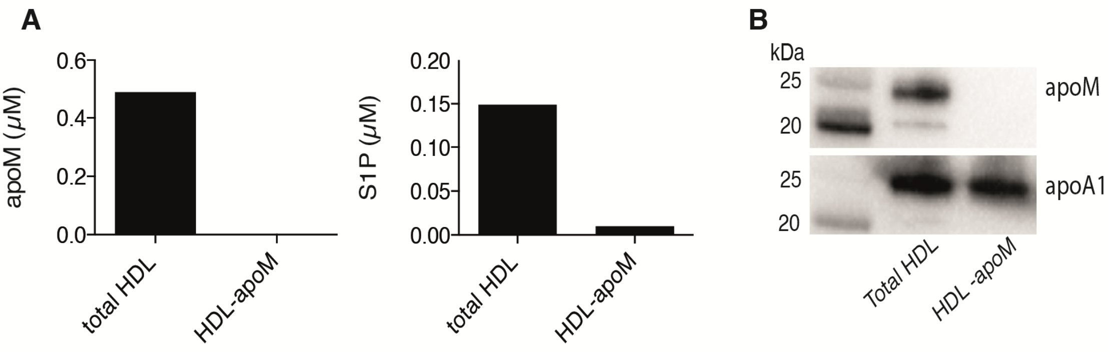


**Fig. S2** S1P in total and apoM-depleted HDL. Levels of apoM and S1P were measured and normalized against apoA1 for analysis (17.3 μM or 0.5 mg/mL apoA1 quantified by ELISA). A), left panel: apoM levels in total HDL or apoM-depleted HDL measured by ELISA; right panel: S1P levels in total HDL or apoM-depleted HDL measured by LC-MS/MS. B), apoM and apoA1 in total HDL or HDL-apoM analysed by western blot

Supplemental figure 3

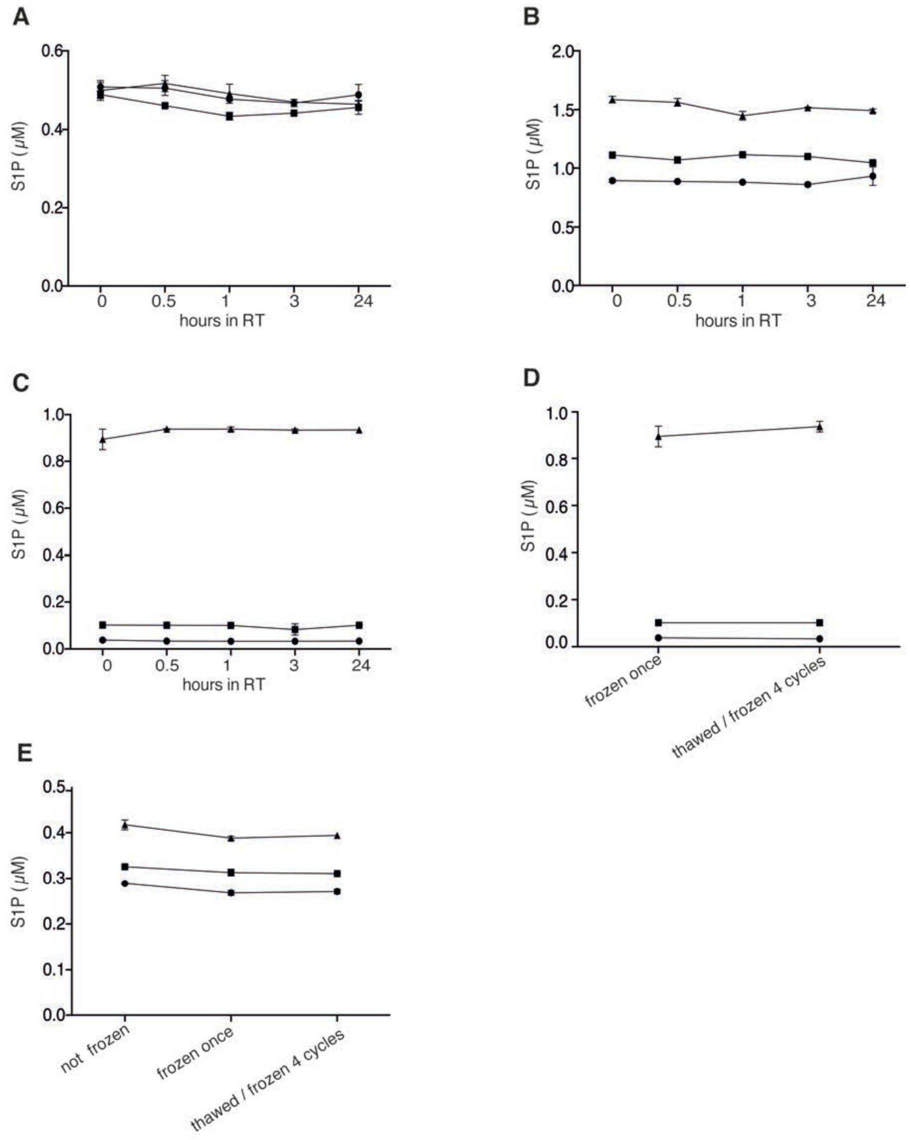


**Fig. S3** Stability of S1P at room temperature and after repeated freeze and thaw cycles. S1P stability at room temperature (RT) was evaluated in (A), citrate plasma (B), serum (C), QC at 0.033, 0.1 and 0.9 μM for 0-24 hours at RT. D-E) Freeze and thaw stability of S1P in D) QC at 0.033, 0.1 and 0.9 μM and E) citrate plasma (n=3). Results are plotted as mean ± SD
